# Minimally Invasive Fassier–Duval Telescopic Rodding of the Lower Limb in Pediatric Osteogenesis Imperfecta: Current Evidence and Implications for Type I (Non-Deforming) Disease

**DOI:** 10.3390/children13091216

**Published:** 2026-09-08

**Authors:** Andreea Moga, Bogdan Popescu, Ruxandra Caragata

**Affiliations:** 1 Department of Pediatric and Orthopaedic Surgery, Carol Davila University of Medicine and Pharmacy, 050474 Bucharest, Romania; andreea.moga@umfcd.ro (A.M.); ruxandra.caragata@umfcd.ro (R.C.); 2 Grigore Alexandrescu Emergency Hospital for Children, 011743 Bucharest, Romania

**Keywords:** osteogenesis imperfecta, Lobstein disease, Fassier–Duval rod, telescopic intramedullary rod, minimally invasive surgery, percutaneous osteotomy, pediatric orthopedics, bone fragility, lower limb

## Abstract

**Highlights:**

**What are the main findings?**

- To our knowledge, this is the first review to synthesize the minimally invasive, percutaneous Fassier–Duval technique—including percutaneous corrective osteotomy and conversion of previously stabilized fractures—and to evaluate its specific relevance to type I (Lobstein) osteogenesis imperfecta, thereby complementing rather than duplicating recent systematic reviews and meta-analyses across study designs.
- Mechanical complications remain common after Fassier–Duval rodding, with implant migration and failure of telescoping predominating; eccentric epiphyseal positioning increases the risk of proximal migration approximately eleven-fold. Evidence specific to type I (non-deforming) osteogenesis imperfecta remains limited, with conclusions largely derived from mixed or more severe phenotypes.

**What are the implications of the main findings?**

- Careful patient selection, meticulous surgical technique, bisphosphonate therapy and multidisciplinary follow-up are essential to optimize long-term outcomes after Fassier–Duval rodding.
- Prospective, phenotype-stratified multicenter studies with standardized outcome reporting are needed to define the role of telescopic fixation in type I (Lobstein) disease.

**Abstract:**

Osteogenesis imperfecta (OI) is the most common heritable bone-fragility disorder, with type I (non-deforming OI) representing its mildest classical phenotype (historically referred to as Lobstein disease). Intramedullary (IM) rodding is essential for managing recurrent long-bone fractures and progressive deformities, with the Fassier–Duval (FD) telescopic rod now being the standard choice. This narrative review, based on a structured search from the seminal 1959 description of IM rodding to 2026, synthesizes the design principles, the minimally invasive technique and the comparative outcomes of FD rodding of the lower limb, with explicit attention to type I disease. Across cohort studies, comparative series and a recent systematic review, telescopic rods consistently achieve lower revision rates and longer implant survival than static or non-telescopic devices, reducing reoperation odds by roughly three-quarters. Overall complication rates nonetheless remain high (commonly 33–55%) and are dominated by implant migration and failure of telescoping. Younger age and eccentric epiphyseal positioning after incomplete deformity correction increase migration risk. Type-I-specific evidence remains limited. A report published under the “Lobstein” designation involved a Sillence type IV patient and therefore does not constitute type-I-specific evidence. Retrospective data nevertheless suggest favorable implant survival with telescopic fixation, but definitive conclusions cannot yet be drawn. Proper technique, adjunctive bisphosphonate therapy and multidisciplinary care remain decisive. Phenotype-stratified prospective data are the principal unmet need.

## 1. Introduction

Osteogenesis imperfecta (OI) is the most common heritable bone-fragility disorder, with a birth prevalence of roughly 1 in 15,000–20,000. It arises from defects in type I collagen, the main structural protein of bone, leaving the skeleton fragile and prone to fractures and deformity [1,2,3]. The clinical spectrum is broad, from a nearly asymptomatic adult to a perinatally lethal phenotype. The mild form has historically been termed Lobstein disease, after Jean-Georges-Chrétien-Frédéric-Martin Lobstein, who described the delayed-onset (“tarda”) form in the early nineteenth century, while in the Sillence classification it is referred to as OI type I [4,5].

For pediatric orthopedic surgeons, the central problem is long-bone fragility, which can lead to recurrent fractures, progressive bowing of the femur and tibia, and failure to achieve or maintain independent ambulation. Intramedullary (IM) rodding is the treatment of choice for preventing fractures and correcting deformities during growth [6,7,8]. The most widely used contemporary implant is the Fassier–Duval (FD) telescopic rod, which elongates with bone growth and is placed through a single antegrade, largely percutaneous entry [9].

Several recent reviews highlight the increasing attention given to this field, although important evidence gaps remain. A narrative review has traced the technical evolution of the implant itself, while quantitative syntheses have pooled outcomes across elongating-rod design, including a meta-analysis of 594 patients spanning four decades and a systematic review of 33 studies involving 861 patients comparing successive rod generations [9,10,11]. Collectively, these studies provide increasing statistical confidence that telescopic rods reduce the need for reoperation compared with non-telescopic or static implants. However, two aspects have received limited attention. First, the minimally invasive, largely percutaneous Fassier–Duval technique, including its integration with percutaneous corrective osteotomy and its use in converting previously fixed fractures, has not been examined as a unifying technical framework. Second, the implications of this approach for the mild type I phenotype remain poorly defined, as published outcome data are dominated by the more severe type III and IV forms, and the Lobstein eponym has not always been used consistently.

Rather than serving as another cross-design outcome meta-analysis, this review provides a technique-oriented synthesis of the minimally invasive Fassier–Duval approach and its adjunctive applications in the lower limb. Based on the broader pediatric OI literature, this review examines the rationale, surgical technique, comparative outcomes, complications and future directions of FD rodding of the femur and tibia, with particular emphasis on delineating what the available evidence does and does not support regarding its use in type I disease.

## 2. Materials and Methods

### 2.1. Search Strategy

This is a narrative (non-systematic) review. A narrative design was chosen deliberately: three quantitative syntheses of elongating-rod outcomes in OI have been recently published [10,11,12], and a further pooled analysis of the same retrospective cohorts would duplicate existing work.

PubMed/MEDLINE, Scopus, Web of Science, Embase and the Cochrane Library were searched, supplemented by SciELO and Google Scholar for regional and open-access literature. Search terms included “osteogenesis imperfecta”, “Lobstein disease”, “Fassier–Duval”, “telescopic rod/nail”, “intramedullary rodding”, “Sofield”, “Bailey-Dubow”, “Sheffield”, “percutaneous”, “minimally invasive” and “osteotomy” combined with AND/OR. The window ran from 1959 with the Sofield–Millar description of IM rod fixation to 3 August 2026, capturing the full technical evolution and the most recent comparative literature.

English-language records were included; pertinent non-English publications (for example Romanian and French) were assessed through translation where they added unique technical or historical content. Reference lists of retrieved articles were hand-searched for further sources.

### 2.2. Selection Criteria

Eligible sources comprised clinical cohorts, comparative studies, technique descriptions, systematic reviews and authoritative disease reviews addressing intramedullary or telescopic rodding in pediatric OI. Adult-only series, non-OI bone-fragility studies (unless directly comparative) and data-free abstracts were excluded. We excluded case reports unless they uniquely demonstrated a relevant technique. The primary exception was a single case report on the percutaneous FD technique under the Lobstein label [13]. Being narrative rather than systematic, the review used no PRISMA flow, meta-analysis or numerical risk-of-bias scoring. Instead, the strength and consistency of the evidence are evaluated qualitatively throughout the text.

## 3. Osteogenesis Imperfecta and Lobstein Disease

The Sillence classification remains widely used clinically despite predating molecular classification. More than 20 OI-associated genes are now recognized, and genotype is increasingly incorporated alongside phenotype. Orthopedically, OI may be viewed broadly as non-deforming, moderately deforming, or severely deforming disease [4,5,14].

Lobstein disease denotes type I OI, is autosomal dominant and usually results from COL1A1 or COL1A2 mutations that reduce the quantity of structurally normal collagen [2,14]. Clinically, it is the mildest common form: near-normal stature, blue sclerae, variable early hearing loss and a fracture tendency that peaks in childhood and wanes after puberty. Deformity, when present, is milder than in types III and IV. The surgical burden is correspondingly lower, which is why the operative literature is dominated by the severe phenotypes, an essential caveat when interpreting outcome data for Lobstein disease [2,5].

Terminological clarification is important in this context. Although Lobstein disease strictly means type I, some authors, including surgical groups reporting FD rodding, use it loosely for OI in general, and it has been applied for type IV patients [13]. As such, verification of the underlying Sillence type is warranted. Regardless of type, recurrent fractures at one site, symptomatic malunion, progressive bowing impairing gait and fractures poorly controlled non-operatively all justify considering IM stabilization, a decision taken within a multidisciplinary framework of bisphosphonate therapy, physical therapy and rehabilitation [3,6,7].

## 4. Evolution of Intramedullary Fixation

**Figure 1:**
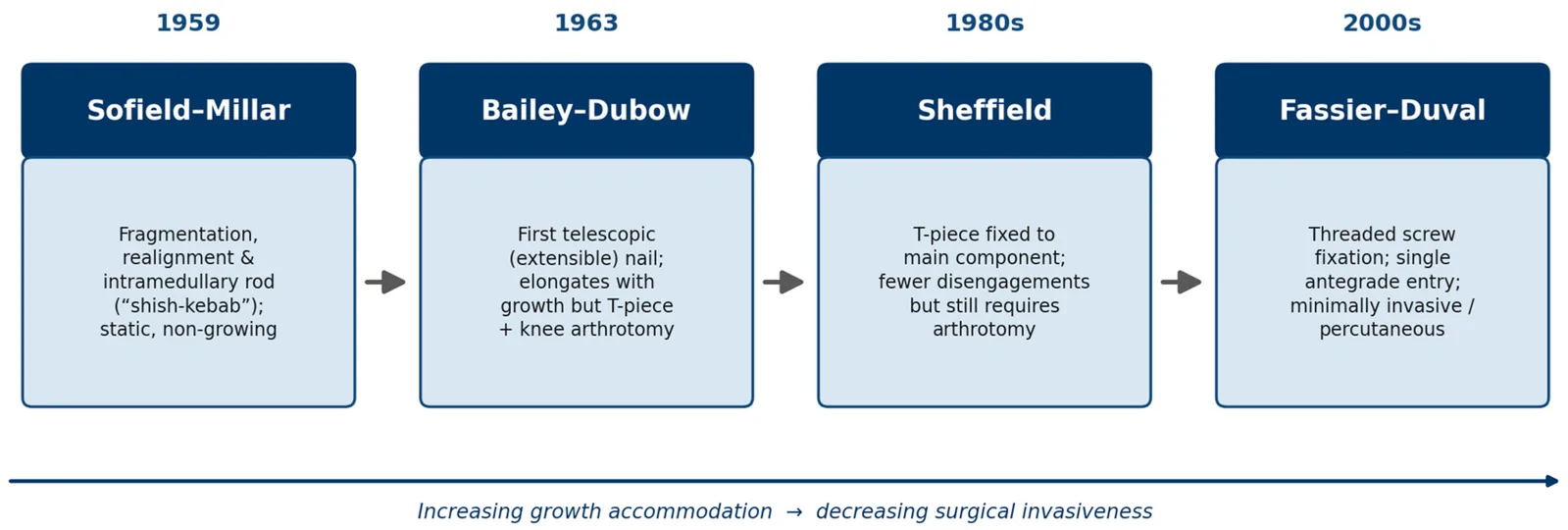
Evolution of intramedullary fixation for long-bone fragility in osteogenesis imperfecta, from the static Sofield–Millar construct to the self-elongating, minimally invasive Fassier–Duval rod. Each generation reduced surgical invasiveness while improving accommodation of skeletal growth.

The modern surgical era began with Sofield and Millar, whose “fragmentation, realignment and intramedullary rod fixation” (the “shish-kebab” operation) straightened bones by threading multiple osteotomy segments onto one rod [8]. Less invasive adaptations using percutaneous or limited osteotomies remain in use [15]. The early static rods (Rush and Steinmann pins) shared one flaw: they do not lengthen. As the child grows, the protected bone outgrows the implant, leaving the metaphysis unsupported and prone to stress risers and recurrent bowing, often requiring repeated surgery [8,16]. The refinements that followed are summarized in Figure 1.

The breakthrough was the lengthening or telescopic rod. Bailey and Dubow introduced a two-part nail whose components slide apart as the bone elongates, letting one implant span the growing bone for years [17]. The Bailey–Dubow device and its later Sheffield modification established the principle but were demanding each was anchored by a transverse “T-piece” requiring a knee arthrotomy and a distal femoral or proximal tibial window [18,19]. Long-term data shows both their value and their cost. The Sheffield system prevented fractures well over a mean nineteen-year follow-up yet caused complications in about a third of segments and reoperation in many patients, while the Bailey–Dubow series reported frequent T-piece disengagement, migration and reoperation [20,21,22]. Paired Rush rods and titanium elastic nails simplified fixation but abandoned true elongation [16].

The Fassier–Duval rod, developed by François Fassier and Pierre-Paul Duval, was engineered to remove these obstacles. Instead of using T-pieces, it is anchored by a threaded distal tip on the male component (screwed into the distal epiphysis) and a proximal one for the female component, so the whole construct is introduced antegrade through a single entry (the greater trochanter or proximal tibial epiphysis) without opening the knee [9,19]. The result is a self-elongating implant inserted through a minimally invasive, largely percutaneous approach, with shorter operative time and less trauma than earlier systems [9].

## 5. The Fassier–Duval Rod: Design and Minimally Invasive Technique

### 5.1. Implant Design

**Figure 2:**
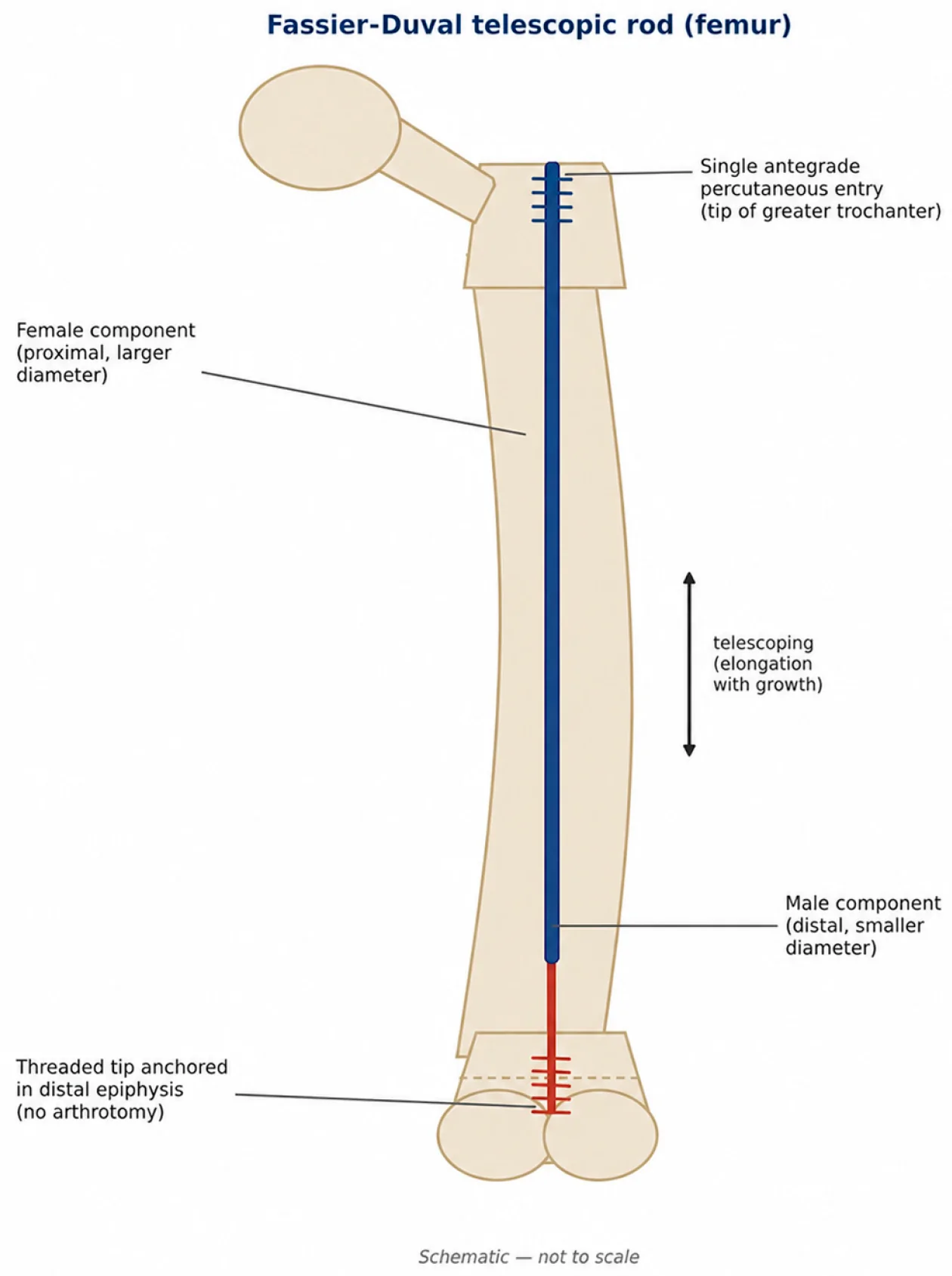
Schematic of the Fassier–Duval telescopic rod in the femur. The larger-diameter female component is anchored proximally at the greater trochanter, and the smaller-diameter male component is screwed into the distal epiphysis; the two telescope apart as the bone lengthens. Insertion is achieved through a single antegrade percutaneous entry without arthrotomy.

The FD system comprises a male and a female component of matched diameter that slide relative to one another as the bone grows (Figure 2). It is available in diameters between 3.2 and 6.4 mm, with lengths for the pediatric femur, tibia and humerus, letting the surgeon match its width to the narrow, gracile OI canal [9]. The male component bears a threaded distal tip for epiphyseal purchase; the female component is fixed proximally. As the physes grow, the two telescope apart, keeping the diaphysis splinted without the need for a longer implant.

### 5.2. Minimally Invasive Percutaneous Insertion

The defining advantage of the FD rod is near-complete percutaneous insertion. In the femur, a single stab incision over the greater trochanter gives the antegrade entry; a guidewire is passed under fluoroscopy, the canal is prepared, and the threaded male rod is advanced to engage the distal femoral epiphysis before the female component is seated proximally [9,19]. Frasier’s account of the operative sequence covering guidewire management, canal preparation and avoidance of the principal intraoperative pitfalls remains the primary technical reference of the procedure [23]. No arthrotomy is needed, and the extensive approach of T-piece designs is avoided. For tibial insertion, infrapatellar or parapatellar approaches are standard; a suprapatellar “retro-patellar” technique through the patellofemoral joint has also been described [24]. Together these approaches reduce soft-tissue stripping, blood loss and operative time, real advantages in children who often need staged, multi-limb surgery.

### 5.3. Combining Percutaneous Rodding with Percutaneous Osteotomies and Fracture Conversion

Where deformity must be corrected, the minimally invasive approach extends to the osteotomies: percutaneous osteotomies are made at the apices of the deformity through small incisions, and the FD nail is passed to realign and splint the segments [25]. Persiani et al. applied this to type III tibial deformities, treating fourteen limbs with the FD nail and percutaneous osteotomies versus eighteen managed openly; the percutaneous group had less pain and faster recovery (about ninety degrees of knee flexion by a mean of approximately 38 days, full weight-bearing by 6 weeks), with comparable patient-reported outcomes, while requiring several distinct learning curves [25].

A related refinement extends the concept to converting a previously operated fracture. Where a femoral fracture had healed with a straight canal around an intramedullary Kirschner wire, the existing wire guided percutaneous introduction of the FD rod without any corrective osteotomy, feasible precisely because the canal was already straight. The authors advised that, when a telescopic rod cannot be used initially, the fracture should be fixed with a thin (≤3 mm) rigid IM implant so that later conversion is minimally traumatic [13].

## 6. Clinical Outcomes and Complications

The main clinical studies of telescopic rodding in OI are compared in Table 1. Rather than recounting each, the following synthesis groups the evidence by theme: implant survival, complication patterns and predictors of failure with the quality of the underlying data.

### 6.1. Implant Survival and Growth Accommodation

Telescopic rods and the FD rod in particular reliably elongate and outlast non-growing implants. Approximately 71% of FD rods demonstrated growth-related telescoping in one representative cohort [26]. The survival advantage over static fixation is substantial, with the hazard of failure for static implants reported to be 13.2 times that of FD rods [27]. In the largest femoral cohort, telescopic rods showed the lowest revision rates in types III and IV (24.8% and 20.9%, respectively), compared with 97.2% and 80.3% for plate fixation, and demonstrated longer implant survival [28]. Nevertheless, growth accommodation does not imply freedom from reintervention; in a longer-term series, revision was required in 53% of patients, most commonly because of growth-related fracture [29]. A separate femoral series with a follow-up of a mean of 9.96 years reported a comparable 36% revision rate at five years, again with fracture as the leading indication [30]. Registry data extend the argument beyond implant survival: among 558 individuals, rodded patients with type III disease had significantly lower annual fracture rates and higher mean mobility scores, although in type IV disease, non-rodded individuals scored higher on nearly all mobility outcomes [31].

### 6.2. Complications and Modes of Failure

The overall complication burden remains high, typically one-third to one-half of rodded segments (about 33–55%) [24,32,33,34,35]. In a single-center cohort of 169 operated limbs in 80 patients followed for a median of 64 months, the overall revision rate was 36.7%, most often for re-fracture (41.9%), recurrent deformity (29%) or rod bending (27.4%); age at surgery for 30 months or less and type IV disease were significant predictors of earlier revision [36]. A single-center seven-year follow-up study of telescopic nailing reached comparable conclusions and prompted a revision of the local management protocol [37].

The dominant failure mechanism is uniform: migration of the male or female component and failure to telescope, with bending, refracture and rotational malalignment less common [32,33]. A two-center analysis mapped these mechanisms anatomically: proximal fixation loss for the femur and distal loss for the tibia with an implant-exchange rate near one-third and a five-year revision-free survival of 63–64% [33]. Incomplete deformity correction can cause eccentric epiphyseal placement, increasing migration risk approximately eleven-fold [34]. Across 66 FD rods followed for a mean of 8.9 years, each millimeter of deviation of the rod from the midline of the distal epiphysis increased the hazard of failure by 7% on the anteroposterior view and by 9% on the lateral view [38]. Bending of the rod is common and is not always benign; among 168 telescopic rods, 46 (27.4%) bent during follow-up, and 27 of these (58.7%) were subsequently revised, although most of the remainder continued to telescope [39].

### 6.3. Predictors of Failure and Quality of the Evidence

Three predictors recur. First is the patient’s very young age: complication risk rises significantly below 5.5 years, although the mechanisms underlying this association remain incompletely defined [32]. In very young children, limited epiphyseal ossification may further compromise reliable threaded fixation. Second is positioning: adequate epiphyseal fixation and a center–center positioning on both radiographic planes are protective, and a strategy built on fixation, center–center positioning and adequate osteotomy has been proposed to cut revision [34,35]. Third is construct stability: supplementary plate fixation lowered the complication rate from 45% to 16% in one comparison, at the cost of added hardware [40]. Where the evidence truly conflicts is in the absolute magnitude of complication and revision rates (roughly 33–55% between series), reflecting inconsistent definitions and differing OI-type case mix, follow-up and thresholds for reintervention. Implant migration or malposition may prompt revision in some series but be observed in others unless associated with recurrent fracture, progressive deformity or functional impairment. Almost all data are Level III-IV retrospective cohorts and case series; there are no randomized comparisons, and outcomes are seldom stratified by Sillence type. These limits temper the precision, though not the direction, of the conclusions [26,29,35].

**Table 1:** Main clinical studies of intramedullary telescopic rodding of the lower limb in pediatric osteogenesis imperfecta discussed in this review. FD, Fassier–Duval; IM, intramedullary; “—”, not reported or not applicable.

Study (Year) [Ref]	Patients (Bones)	OI Type	Implant	Follow-Up	Compl.	Revision	Key Findings
Nicolaou 2011 [22]	cohort	Mixed	Sheffield rod	~19 y (mean)	~29%	~43%	Effective long-term fracture prevention; substantial reoperation rate over two decades.
Birke 2011 [41]	15 (24 rods)	Mixed (OI subgroup)	Fassier–Duval	≥1 y	40%	13%	First FD series; complications chiefly proximal migration and incomplete telescoping.
Azzam 2018 [29]	58 (179 rods)	Mixed	Fassier–Duval	>4 y (mean)	—	53%	Revision at mean ~52 mo, mostly growth-related; nonunion in 14.5%.
Rosemberg 2018 [30]	21 (52 femoral rods)	I, III, IV	Telescopic IM rod	9.96 y (mean)	—	36% at 5 y	Fracture the leading cause of revision, then migration and infection.
Shin 2018 [42]	26 vs. 31 tibiae	OI	Dual vs. single-interlocking rod	5.3 y/9.6 y (mean)	54% vs. 81%	—	Dual interlocking reduced refracture and system-related complications.
Persiani 2019 [25]	14 tibiae (vs. 18 open)	III	FD + percutaneous osteotomy	—	—	—	Less pain and faster recovery than open osteotomy; multiple learning curves.
Spahn 2019 [27]	21 (64 limbs)	Mixed	FD vs. static	—	—	—	Static-implant failure hazard 13.2× that of FD; 7.8× more total surgery.
Bhaskar 2019 [43]	21	OI	IM rodding	—	—	—	Rodding improved ambulation and reduced refracture.
Cox 2020 [33]	34 (72 bones)	OI	FD (single-entry)	—	—	~33% exchange	Proximal (femur)/distal (tibia) fixation loss; 5 y revision-free survival 63–64%.
Holmes 2020 [38]	13 (66 FD rods)	OI	FD	8.9 y (mean)	—	—	Hazard of failure rose 7%/mm AP and 9%/mm lateral epiphyseal deviation.
Rodriguez Celin 2020 [31]	558 individuals	I, III, IV	Expanding vs. non-expanding	registry	—	—	Type III: fewer fractures/year and higher mobility scores when rodded.
Musielak 2021 [32]	19 (58 segments)	Mostly III	Fassier–Duval	4.4 y (median)	44.8%	—	Migration predominant; complication risk rises below age 5.5 y.
Suresh 2021 [44]	24 FD rods; 21 tibia, 3 femur	OI	FD with single interlocking pin	7.2 y (mean)	—	58%	Pin back-out drove 42% of revisions; bending the pin tip reduced it.
Hung/Liu 2022 [35]	7 (20 bones)	III/IV	Fassier–Duval	—	—	55%	Depth of purchase, center–center position and adequate osteotomy reduce revision.
Emet 2023 [40]	40 (61 limbs)	Mild–severe	IM vs. IM + plate	4.4 y (mean)	45% vs. 16%	—	Supplementary plating markedly lowers complications vs. IM fixation alone.
Fralinger 2023 [39]	43 (168 rods)	Severe vs. non-severe	Telescopic rods, lower limb	≥1 y	27.4% bent	58.7% of bent rods	Most unrevised bent rods continue to telescope.
Goiano 2023 [26]	15 (21 rods)	I, III, IV	Fassier–Duval	5.2 y (mean)	33.3%	—	71% of rods telescoped with growth; cohort included 3 type I patients (4 rods), with a mean telescoping increase of 7.0% in the type I subgroup.
Yang 2023 [28]	541 (783 femora; 322 FD)	I, III, IV	FD vs. plate/elastic/non-elongating	up to 20 y	Refracture 15.5% (FD)	Type I: 19.1%; III: 24.8%; IV: 20.9% (FD)	Type I subgroup: 97 procedures, including 47 FD rods; FD revision rate was 19.1% (9/47), with longer implant survival than plates despite no significant revision-rate difference (*p* = 0.063).
Li 2024 [45]	388 femoral procedures	I, III, IV	Telescopic vs. elastic nail/plate/K-wire	—	30% vs. 87.7%/90.1%/96.8%	—	Telescopic rod had the lowest implant-related complication rate.
Nguyen 2025 [46]	52 (119 bones)	OI	Telescopic vs. static	13.1 y (mean)	—	2.2 vs. 3.3 procedures/bone	Telescopic rods survived 5.8 y vs. 4 y and needed fewer procedures.
Tangadulrat 2026 [36]	80 (169 limbs)	OI (type IV a risk factor)	FD	64 mo (median)	—	36.7%	Age ≤ 30 mo and type IV predicted earlier revision.
Yoshida 2026 [12]	1711 (2459 bones); 36 studies	All	Telescopic vs. non-telescopic	5.6/6.2 y	45% vs. 56%	24% vs. 56%	Systematic review: telescopic rods reduce reoperation odds ~76% (OR 0.24).
Bălănescu 2013 [13]	1 (case report)	IV (reported as ‘Lobstein’)	FD percutaneous conversion	—	—	—	Percutaneous conversion of previous K-wire fixation to FD without corrective osteotomy; technical evidence only, not type-I-specific outcome evidence.

## 7. Comparative Evidence and Remaining Controversies

The comparative literature conveys a consistent overall message while leaving specific questions unresolved. The most comprehensive synthesis to date, a 2026 systematic review including 36 studies (1711 patients, 2459 bones), found no statistically significant difference in the overall risk of complications when comparing telescopic and non-telescopic rods (about 45% versus 56%) but revealed a substantially lower need for reoperation with telescopic devices (roughly 24% versus 56%; odds ratio near 0.24, about a 76% relative reduction) [12]. Two further recent syntheses reinforce this pattern: a meta-analysis of 594 patients treated over four decades confirmed that complications of elongating rods remain common despite their growth-accommodating advantage [10], while a systematic review of successive rod generations reported design-specific complication and revision rates—37.1% and 30.5% for Fassier–Duval rods, with 74% telescoping success—and suggested that the newest interlocking and distal-female designs may lower these rates further, albeit with shorter follow-up [11]. Similarly, El-Adl et al. reported lower reoperation rates with telescopic rods, while both Spahn’s comparison with static implants and Yang’s large femoral cohort demonstrated findings in the same direction [27,28,47]. The longest follow-up available points the same way: across 119 bones in 52 patients followed for a mean of 13.1 years, telescopic rods achieved a mean survival of 5.8 years and required 2.2 procedures per bone, against 4 years and 3.3 procedures for static rods [46]. In a series of 388 femoral procedures stratified by implant, implant-related complications occurred in 30% of telescopic rods, against 87.7% for elastic nails, 90.1% for plates and 96.8% for Kirschner wires [45]. Although the magnitude of benefit and its applicability to individual clinical scenarios remain open to further investigation, the overall evidence consistently favors telescopic fixation in terms of reducing the need for repeat surgery.

The remaining controversies relate primarily to the technical aspects of the treatment rather than to the underlying principle of telescopic fixation. One unresolved issue is whether supplementary plate fixation should routinely accompany telescopic rods. Adjunctive plating may reduce migration and early complications, but its use is limited by age and bone geometry. In small OI bones, screw purchase may be restricted by the intramedullary implant, an issue insufficiently addressed in the current literature [40]. A second unresolved issue is the fact that optimal timing for surgery remains uncertain. While earlier rodding may protect the developing limb before progressive deformity occurs, intervention before 5.5 years of age has been associated with higher complication rates [32]. A third unresolved question concerns distal anchorage. Interlocking designs have been proposed to address the loss of epiphyseal purchase that dominates FD failure, and a dual-interlocking tibial rod produced fewer refractures and system-related complications than a single-interlocking construct (54% of 26 tibiae vs. 81% of 31) [42]. In a series of 24 FD rods carrying a single interlocking pin, 58% required revision, with pin back-out accounting for 42%, so any benefit appears to depend on the specific construct rather than on interlocking as a principle [44]. Finally, the lack of standardized definitions for postoperative complications contributes substantially to heterogeneity across published studies, limiting the validity of pooled analysis and comparisons between series [12]. Future multicenter registries incorporating standardized outcome definitions and phenotype-stratified analyses will be essential to address these remaining questions and refine surgical decision-making.

## 8. Implications for Type I (Lobstein) Disease

Clinical evidence specific to type I OI remains limited. In the largest comparative femoral series, Yang et al. reported 97 type I procedures, including 47 treated with Fassier–Duval rods [28]. The revision rate in the FD group was 19.1% (9/47). Although revision rates did not differ significantly between implant types (*p* = 0.063), telescopic rods showed significantly longer implant survival than plate fixation (*p* = 0.001). Goiano et al. also reported three type I patients treated with four FD rods, with preserved growth-related telescoping, although the small sample precludes firm phenotype-specific conclusions [26]. Bălănescu et al. should be interpreted separately [13]. Although the report used the term “Lobstein disease,” the patient was classified as Sillence type IV. It therefore supports the technical feasibility of percutaneous conversion to an FD rod but does not provide type-I-specific clinical evidence. Beyond the limited clinical evidence available, the biomechanical characteristics of type I disease may also influence the technical performance of FD fixation. Compared with types III and IV, type I disease is generally associated with milder deformity and more favorable bone morphology, factors that may facilitate percutaneous implantation; however, whether these characteristics translate into lower complication or revision rates has not been established. Nevertheless, these assumptions remain speculative in the absence of phenotype-specific comparative data. Regardless of the severity of the disease, the fundamental surgical principles of stable fixation, center–center rod positioning and correction of residual deformity remain essential to optimize implant longevity and reduce mechanical failure [34,35]. Operative indication for type I is less frequent, and as such the decision to intervene should be individualized, balancing fracture risk, progressive deformity, functional impairment and expected growth. In carefully selected patients, the durability of telescopic fixation and its reduced likelihood of requiring revision surgery may represent particular advantages. Prospective studies reporting outcomes separately for patients with type I are needed to validate these assumptions and better define optimal management [3,7].

## 9. Adjunctive Medical Therapy and Multidisciplinary Care

Intramedullary rodding should be considered as a component of a comprehensive multidisciplinary treatment strategy for osteogenesis imperfecta. Cyclical intravenous bisphosphonate therapy, initially established with pamidronate and now frequently administered as zoledronic acid, has been shown to increase bone mineral density, reduce fracture incidence and improve functional outcomes in children with moderate to severe disease, making it the standard medical adjunct to surgical management [48]. Randomized trials have also demonstrated that oral bisphosphonates, such as alendronate, improve bone mineral density, although their effect on fracture reduction appears more modest [49].

The interaction between bisphosphonate therapy and surgery warrants consideration. While fracture healing is generally preserved, delayed osteotomy healing has been reported in children receiving bisphosphonates, with potential implications for postoperative rehabilitation, weight-bearing protocols and radiographic follow-up after FD rodding. In the largest series addressing this question, delayed healing followed 42% of 114 osteotomies performed under a revised protocol—a four-month bisphosphonate-free interval after osteotomy, zoledronic acid and use of an osteotome rather than a power saw, compared with 72% of 147 osteotomies managed under the previous approach [50]. Accordingly, successful treatment depends not only on appropriate implant selection and surgical technique but also on close coordination between metabolic bone specialists, orthopedic surgeons, physiotherapists and rehabilitation teams to maximize functional outcomes and long-term skeletal health [14,51]. Patient-reported data underline the same point: in a cohort of 15 children assessed after telescopic rodding, acute and chronic pain impaired quality of life and interfered with self-care, with the poorest scores among those living in rural areas [52].

## 10. Future Perspectives

Two questions will shape the future of telescopic fixation in OI: why telescopic rods continue to fail and how advances in technology may reduce this risk. Failure reflects both biological and mechanical factors. The osteoporotic epiphysis and thin cortex that are characteristic of OI provide limited purchase for threaded rod ends, predisposing to loosening even after technically satisfactory implantation. Because fixation relies on a relatively small threaded interface at each extremity of the implant, even minor deviations from optimal center–center positioning can concentrate mechanical stress and promote migration or failure [33,34]. Retrieval analysis supports this mechanically. Across 25 explanted telescopic rods the female component was less hard than the male (Vickers HV0.3 313 vs. 406) and showed a higher wear coefficient, and abrasive wear, scratches and wear debris were identified in all rods, indicating that material behaviors contribute to late failure alongside bone quality and implant position [53]. These observations suggest that, although disease severity contributes to implant performance, meticulous surgical technique and accurate implant positioning remain modifiable determinants of outcome.

Several technological developments may further improve fixation. Implant refinements, including enhanced epiphyseal anchorage, interlocking or “rescue” constructs, improved materials and lower-cost designs to expand global accessibility, seek to reduce mechanical failure while preserving the advantages of telescopic growth accommodation. Advances in intraoperative navigation and robotic-assisted surgery may improve the reproducibility of optimal implant positioning, directly addressing one of the principal mechanisms of rod migration. Similarly, three-dimensional CT-based planning, patient-specific instrumentation and computer-assisted osteotomy guides have the potential to improve deformity correction and optimize implant trajectories in the narrow intramedullary canals typical of children with OI [9,35]. In parallel, emerging medical therapies, including next-generation antiresorptive agents, anabolic therapies, and gene-targeted approaches, may further reduce fracture burden and the need for repeated surgical intervention [14].

Future research should move beyond heterogeneous retrospective series toward prospective multicenter studies with phenotype-specific analyses and standardized endpoints. Reporting should include implant survival, revision indications, mechanical complications, functional outcomes and patient-reported measures, allowing more reliable comparison across techniques and OI phenotypes. Such studies would also provide much-needed outcome data specific to type I (Lobstein) disease.

## 11. Limitations

This review has several limitations. Although it was based on a structured literature search, it is a narrative rather than a systematic review and did not include PRISMA-guided study selection, metanalysis or formal quantitative assessment of risk of bias. Consequently, selection and reporting bias cannot be excluded. The available evidence consists predominantly of retrospective cohort studies and case series, with heterogeneous outcome definitions, relatively small sample sizes and variable durations of follow-up. Furthermore, few studies stratify outcomes according to Sillence classification, resulting in limited evidence specific to patients with type I (Lobstein) disease [7,14]. Interpretation is further complicated by the evolution of surgical instrumentation, adjunctive medical therapies and operative expertise over the several decades encompassed by the published literature. Accordingly, the conclusions presented here should be viewed as the best available synthesis of predominantly Level III–IV evidence rather than as definitive estimates of treatment effect.

## 12. Conclusions

The minimally invasive Fassier–Duval telescopic rod has become a widely used intramedullary implant for long-bone stabilization in skeletally immature patients with osteogenesis imperfecta, including selected patients with type I (Lobstein) disease. Its self-elongating design and predominantly percutaneous, single-entry insertion address many of the limitations of static rods and earlier telescopic systems, while allowing correction of deformity through minimally invasive osteotomies when indicated. Comparative evidence consistently demonstrates lower revision rates and longer implant survival than with non-telescopic or static implants, although complications related to rod migration, failure of telescoping and fixation remain common, particularly in younger children and when implant positioning is suboptimal. Although type-I-specific evidence remains limited, available phenotype-stratified and mixed-cohort data support the feasibility of Fassier–Duval fixation in selected patients with mild OI. Current evidence remains insufficient to establish whether revision rates, implant survival or complication profiles differ significantly from those observed in more severe phenotypes, and prospective phenotype-stratified studies are required. Careful patient selection, meticulous surgical technique, integration of medical therapy and multidisciplinary follow-up remain essential to optimizing long-term outcomes. Prospective, phenotype-stratified studies with standardized outcome reporting are needed to define the role of telescopic fixation across the full spectrum of osteogenesis imperfecta.

## Data Availability

No new data were created or analyzed in this study. Data sharing is not applicable to this article.
